# Particle-Based Numerical Simulation Study of Solid Particle Erosion of Ductile Materials Leading to an Erosion Model, Including the Particle Shape Effect

**DOI:** 10.3390/ma15010286

**Published:** 2021-12-31

**Authors:** Shoya Mohseni-Mofidi, Eric Drescher, Harald Kruggel-Emden, Matthias Teschner, Claas Bierwisch

**Affiliations:** 1 Fraunhofer IWM, Wöhlerstraße 11, 79108 Freiburg, Germany; claas.bierwisch@iwm.fraunhofer.de; 2 Department of Microsystems Engineering, University of Freiburg, Georges-Köhler-Allee 103, 79110 Freiburg, Germany; 3 Chair of Mechanical Process Engineering and Solids Processing (MVTA), Technical University of Berlin, Ernst-Reuter-Platz 1, 10587 Berlin, Germany; e.drescher@tu-berlin.de (E.D.); kruggel-emden@tu-berlin.de (H.K.-E.); 4 Department of Computer Science, University of Freiburg, Georges-Köhler-Allee 106, 79110 Freiburg, Germany; teschner@informatik.uni-freiburg.de

**Keywords:** solid particle erosion, particle shape, contact models, smoothed particles hydrodynamics

## Abstract

Solid particle erosion inevitably occurs if a gas–solid or liquid–solid mixture is in contact with a surface, e.g., in pneumatic conveyors. Having a good understanding of this complex phenomenon enables one to reduce the maintenance costs in several industrial applications by designing components that have longer lifetimes. In this paper, we propose a methodology to numerically investigate erosion behavior of ductile materials. We employ smoothed particle hydrodynamics that can easily deal with large deformations and fractures as a truly meshless method. In addition, a new contact model was developed in order to robustly handle contacts around sharp corners of the solid particles. The numerical predictions of erosion are compared with experiments for stainless steel AISI 304, showing that we are able to properly predict the erosion behavior as a function of impact angle. We present a powerful tool to conveniently study the effect of important parameters, such as solid particle shapes, which are not simple to study in experiments. Using the methodology, we study the effect of a solid particle shape and conclude that, in addition to angularity, aspect ratio also plays an important role by increasing the probability of the solid particles to rotate after impact. Finally, we are able to extend a widely used erosion model by a term that considers a solid particle shape.

## 1. Introduction

Solid particle erosion (SPE) is a repetitive process that causes material removal from a surface due to solid particle impacts. It happens wherever solid particles, e.g., sands, metallic particles, and foods, are carried in a conveying system by liquids or gases. Several industries, such as energy producers, pharmaceuticals, and chemical engineering, employ conveying systems. SPE may cost these industries a fortune by damaging conveyors, which requires regular maintenance and sometimes bring about substantial harm to the system, due to total failure of parts. Therefore, by estimating erosion in conveying systems, one can optimize operating conditions in order to attain better designs and parts, for longer lifetimes, and to reduce maintenance and production costs.

Predicting the SPE of conveyors mainly involves two fields of studies. First, the study of the mechanism of solid particle impacts. Second, the study of the dynamics of the particles that is defined by the geometry of conveyors and hydrodynamics of carrier fluids. Commonly, these two studies are unified by using an erosion model in a coupling between computational fluid dynamics (CFD) and the discrete element method (DEM). Erosion models are theoretical or empirical models that define the surface mass loss due to a single particle impact, depending mainly on the impact velocity and the impact angle of particles that are calculated by a CFD–DEM method. On the one hand, Messa et al. [1] provided a thorough overview of numerical methods employed to model slurry flows in pipelines as well as discussions about different fluid–solid coupling scenarios. On the other hand, Parsi et al. [2] offered a comprehensive review of erosion models that are used in CFD–DEM simulations to model erosions in oil and gas pipelines. All erosion models, regardless of being theoretical or empirical, have fitting parameters that are typically calibrated by using experimental data. To obtain the required information, blasting tests are commonly employed where a surface specimen is eroded by a jet of gas–solid or liquid–solid mixture.

*Theoretical erosion models:* In the seminal works of Finnie [3] and Bitter [4,5], the authors experimentally investigated the surface erosion of ductile and brittle materials. Based on their findings, they ascribed the material removal to the cutting mechanism at shallow impact angles and the deformation mechanism at large angles. Moreover, they also developed the very first theoretical erosion models. Although Finnie’s model produces good results at an angle of the maximum erosion, it poorly estimates erosion at other angles, especially at large angles. Bitter’s model [4,5] could not express the surface erosion as a function of material properties, but instead introduced two individual scaling factors for each erosion mechanism. Hutchings [6] and Sundararajan and Shewmon [7] developed erosion models for normal impacts, depending on material properties, namely, hardness and ductility by using a criterion of critical plastic strain. To define the criterion, Hutchings [6] suggested that the material removal is cased by the low cycle fatigue, whereas Sundararajan and Shewmon [7] assumed that it happens by lip formations due to deformation localization. Like many other researchers, Huang et al. [8] applied the idea of two erosion mechanisms, namely cutting and deformation, suggested by Bitter [4,5]. They estimated the volume loss due to deformation by solving the equation of motion for a spherical particle in a normal direction and applying a critical strain criterion used by Hutchings [6]. Additionally, they defined the volume loss due to cutting, assuming that the cutting profile varies depending on the material properties and the shape of the particle’s edge. Arabnejad et al. [9] employed erosion models proposed by Finnie [3] and Bitter [4,5] to determine the volume loss due to deformation and cutting, respectively, and then combined them, using empirical coefficients, to form a generalized semi-mechanistic erosion model. Following a different path, Uzi and Levy [10] associated the surface erosion due to cutting and deformation to the energy loss in vertical and tangential directions, respectively, during an impact, and proposed a model to estimate the erosion normalized by erosion at a normal angle. They defined the energy losses as fractions of the kinetic energy, based on the material hardness, surface damping coefficient, and coefficient of restitution in normal and tangential directions. The authors further improved the model in Uzi and Levy [11] by introducing a new model in order to calculate erosion and incorporate the effect of particle size as well.

*Empirical erosion models:* In addition to theoretical erosion models, some mentioned above, many researchers proposed empirical models, which are mainly dependent on quantities that can be experimentally measured, such as impact velocity, impact angle, particle size, and material hardness. It is very convenient to adopt these models in numerical studies since they typically do not have complex forms. Erosion can then be calculated only by knowing the impact velocity and angle. Oka and Yoshida [12] and Oka et al. [13] systematically studied the surface erosion of several metals for different impact velocities with particles of different types and sizes. They proposed an erosion model that includes an impact angle dependent function, which scales the erosion at a normal impact that depends on impact velocity, particle size, and material hardness. Zhang et al. [14] introduced the erosion model proposed by the Erosion/Corrosion Research Center (E/CRC) where dependency of erosion on impact angle and velocity is defined by a fifth order polynomial and a power law, respectively. The authors carried out simulations of erosion of Inconel 718 in a 90° blasting test and a 90° elbow by using the E/CRC model and Oka’s model. It was shown that the obtained numerical results for both models are in good agreement with the experiments. Vieira et al. [15] investigated the erosion of stainless steel 316 by sand particles and proposed an erosion model similar to Oka’s model, where the angle dependent function of Oka’s model is modified and the effect of particle shape is considered by a sharpness coefficient. Moreover, they employed a CFD code that has a particle-tracking option in order to validate the model by simulating the erosion in elbows. Very recently, Banazadeh-Neishabouri and Shirazi [16] developed two erosion models for SPE of fiberglass-reinforced plastic, by modifying the Oka and Zhang erosion models that were mainly proposed for metals. Their results showed that the developed models can lead to good predictions of SPE, especially for relatively low impact velocities.

*Numerical studies:* SPE is a very complex phenomenon that involves different mechanisms and depends on several factors. Researchers commonly make assumptions to simplify SPE in order to be able to develop theoretical models. Therefore, these models always include some coefficients, which must be tuned properly in order for erosion predictions to match experiments. Moreover, since it is too costly and sometimes impossible to investigate all influential parameters in experiments, the effects of some parameters, such as particle shape and size, is simply modeled by a scaling factor in empirical erosion models. That being said, numerical simulations can be very helpful to study the mechanisms and examine the parameters that are neglected in theoretical and experimental works. During the past two decades, numerical methods, such as finite element method (FEM), smoothed particle hydrodynamics (SPH), and finite volume particle method (FVPM) have been employed to study the mechanisms of the material removal and surface deformation, due to particle impacts and, hence, provide a deeper understanding of SPE. On the one hand, some numerical studies were only dedicated to simulations of the impact of a single particle. Molinari and Ortiz [17] used FEM to model the impact of a deformable spherical particle and studied the deformation of the particle and the temperature rise of the surface over a wide range of impact velocities. Li et al. [18] studied the single impact of spherical particles on steel samples with different impact angles at ultrahigh velocities by using FEM. In addition to plasticity, they employed a damage model in order to not only examine the kinetic energy loss during an impact, but also to identify the modes of material failure. Additionally, Dong et al. [19] and Dong et al. [20] employed SPH to simulate the surface material removal due to single impacts of angular particles for a wide range of impact angles and particle orientations. They showed that the energy absorption by the surface varies not only as a function of particle angularity, but also as a function of the orientation angle and the centroid offset angle that determine whether a particle rotates forward or backward. On the other hand, several researchers carried out simulations of multiple impacts in order to model the accumulation of the surface damage due to overlapping impacts. It was shown by ElTobgy et al. [21] that SPE cannot be accurately estimated by modeling a single particle impact. Takaffoli and Papini [22] employed SPH to model SPE of aluminum 6061-T6 by multiple overlapping angular particle impacts. They successfully predicted the surface erosion behavior and identified fracture modes resulting from the interaction of the lip of craters with the successive impacts. Li et al. [23] studied multiple high velocity impacts of spherical particles by using FEM. They comprehensively investigated thermal effects, e.g., thermal softening and diffusion, and overlapping impacts on the surface material failure. Liu et al. [24] investigated the effect of the particle shape on the erosion behavior of several ductile materials by using FEM. They employed a methodology similar to Woytowitz and Richman [25], where 100 particles hit a surface arranged in 10 parcels, each of which includes 10 particles positioned randomly. By using uniform polyhedral particles, they were able to predict the erosion behavior obtained by experiments for irregularly shaped particles. Leguizamón et al. [26] employed FVPM to study the effect of particle shape and elasticity on SPE for oxygen-free copper and martensitic stainless steel 13Cr-4Ni. They developed an algorithm to generate realistic 3D particles. They concluded that the deformation of particles may have considerable effect on erosion and showed that sharp particles can produce much higher erosion than spherical particles, especially at low impact angles.

In this paper, we aim to numerically predict erosion behavior of ductile materials. For this reason, we employ SPH to model SPE of stainless steel AISI 304. Interactions between the carrier fluid and solid grains are disregarded which is a valid assumption when the carrier fluid is air and the solid grains are large and, thus, the momentum of the carrier fluid can be neglected in comparison to the momentum of the grains. There are two distinct numerical methodologies to properly model surface erosion behavior and material removal mechanisms. In the first methodology similar to Liu et al. [24], particles are randomly positioned over a rather wide range and hit a surface in parcels. Particles of each parcel do not interact with each other are placed at least 0.6 times the particle radius apart and impact the surface simultaneously. According to Li et al. [23], simultaneous impacts may impose constraints on deforming regions entrapped between close impacts and, consequently, change thermal softening behavior and damaging of the surface. As employed by Leguizamón et al. [26], the second methodology consists of random single impacts that hit the surface one after the other within a small region, typically one particle diameter wide. In this study, we use the second methodology, since it does not suffer from the aforementioned drawback. However, Leguizamón et al. [26] could not properly predict erosion behavior in terms of impact angle. Moreover, their calculated erosions showed almost the same behavior for angular and spherical particles. Therefore, we set out to improve the methodology in order to get reliable predictions of erosion at different impact angles. To obtain this goal, we ensure that the simulated impacts can statistically represent all possible impacts. Moreover, we employ 2D models and show that, although 2D simulations can not quantitatively predict erosion, they provide a very powerful tool to study the effect of important parameters, such as particle shape. It is worth mentioning that the methodology can easily be extended to 3D simulations. In addition, in order to properly model contacts between particles of different shapes a new method was developed that extrapolates a particle’s surface normals onto the surface in a straightforward manner and, hence, robustly handles contact detections and contact force calculations around sharp corners. Ultimately, we are able the extend the erosion model by Oka et al. [13] by dimensionless factors, which take the grain shape, in the form of angularity and the aspect ratio, into account.

## 2. Smoothed Particle Hydrodynamics

In this paper, smoothed particle hydrodynamics is employed to model surface erosion caused by angular solid particle impacts. For the sake of clarity, in what follows, solid particles are referred to as grains and SPH particles are simply mentioned as particles. Material domains are discretized by particles that, as material points, carry physical quantities, such as density, mass, velocity, and stress during simulations. Using SPH, conservation equations of mass, momentum, and energy are solved by using SPH approximations that are defined for a field function $A(\vec{x})$ and its derivatives at particle *i* as

(1) $$\;\langle A({\vec{x}}_{i})\rangle =\sum_{j=1}^{N}\frac{m_{j}}{\rho_{j}}A\left({\vec{x}}_{j}\right)\;W\left(x_{ij},h\right),$$

(2) $$\;\langle \nabla ⊕A({\vec{x}}_{i})\rangle =\sum_{j=1}^{N}\frac{m_{j}}{\rho_{j}}\left(A\left({\vec{x}}_{j}\right)-A\left({\vec{x}}_{i}\right)\right)⊕\nabla W\left(x_{ij},h\right),$$

(3) $$\;\langle \nabla ⊕A({\vec{x}}_{i})\rangle =\rho_{i}\sum_{j=1}^{N}m_{j}\left(\frac{A({\vec{x}}_{j})}{{\rho_{j}}^{2}}+\frac{A({\vec{x}}_{i})}{{\rho_{i}}^{2}}\right)⊕\nabla W\left(x_{ij},h\right),$$

where *m* and ρ are the mass and density of a particle, respectively. The $\langle \ldots \rangle$ symbol represents the approximation of a quantity and it is omitted in the rest of the paper for the sake of simplicity. In addition, ⊕ represents the type of the operator Δ, namely gradient, divergence and tensor product. Equation (2) is used to approximate the velocity divergence in the mass conservation equation ensuring the zero completeness of the approximation, whereas Equation (3) is applied to the stress divergence of the momentum conservation equations to guarantee the local conservation of momentum between particle pairs [27]. *W* is a kernel function that weighs the contribution of each particle in an SPH approximation at a given particle position, thereby generating pseudo-connectivities among particles. *W* is a positive monotonically decreasing function depending on the distance between particles *i* and *j*, $x_{ij}=\left|{\vec{x}}_{i}-{\vec{x}}_{j}\right|$, and the smoothing length of the kernel function, *h*, that is proportional to the initial particle spacing, Δx, and is here equal to 1.3Δx. Hence, SPH approximations for the particle *i* only involve the contributions of the *N* supporting particles for which the kernel function is non-zero. In this work, we employ the quintic spline kernel function expressed as

(4) $$\;\;W\;\left(x_{ij},h\right)=\frac{k}{h^{d}}\left\{\begin{array}{cc} {\left(3-q\right)}^{5}-6{\left(2-q\right)}^{5}+15{\left(1-q\right)}^{5}, & 0\leq q<1,\\ {\left(3-q\right)}^{5}-6{\left(2-q\right)}^{5}, & 1\leq q<2,\\ {\left(3-q\right)}^{5}, & 2\leq q<3,\\ 0, & q\geq 3, \end{array}\right.$$

where *d* is the number of dimensions of the problem, $q=x_{ij}/h$ and *k* is the normalization factor that is equal to 7/(478π) for d=2 and 1/(120π) for d=3. The following discretized mass and momentum conservation equations are employed, which are corrected and stabilized in order to accurately predict the behavior of a surface under repetitive impacts of solid grains,

(5) $$\begin{array}{cc} \;\frac{d\rho_{i}}{dt} & =\rho_{i}\sum_{j=1}^{N}\frac{m_{j}}{\rho_{j}}{\vec{v}}_{ij}\;\cdot \;\tilde{\nabla }W_{ij}, \end{array}$$

(6) $$\begin{array}{cc} \;\frac{d{\vec{v}}_{i}}{dt} & =\sum_{j=1}^{N}m_{j}\left(\frac{S_{i}}{\rho_{i}^{2}}\;\cdot \;\hat{\nabla }W_{ij}-\frac{S_{j}}{\rho_{j}^{2}}\;\cdot \;\hat{\nabla }W_{ji}\right)+\frac{1}{m_{i}}\sum_{j=1}^{N}{\vec{f}}_{ij}{\;}^{\mathrm{HG}}+\frac{1}{m_{i}}{\vec{f}}_{i}{\;}^{\mathrm{cont}}. \end{array}$$

In the mass conservation equation, Equation (5), ${\vec{v}}_{ij}$ is the relative velocity of particles *i* and *j* and $\tilde{\nabla }W_{ij}$ is the corrected kernel gradient that is proposed by Bonet and Lok [28] to ensure the first-order completeness defined by

(7) $$\;\tilde{\nabla }W_{ij}=L_{i}\;\nabla W_{ij},$$

where $L_{i}$ is the correction matrix for particle *i* expressed by

(8) $$\;L_{i}={\left(\sum_{j=1}^{N}\frac{m_{j}}{\rho_{j}}{\vec{x}}_{ij}⊗\nabla W_{ij}\right)}^{-1},$$

where ${\vec{x}}_{ij}={\vec{x}}_{i}-{\vec{x}}_{j}$ is the distance vector between particles *i* and *j*. However, the momentum conservation equation, Equation (6), is discretized by using the following normalized kernel gradient that guarantees the zero-order completeness of the stress divergence especially close to the boundaries,

(9) $$\;\hat{\nabla }W_{ij}=\frac{\nabla W_{ij}}{\sum_{j=1}^{N}\frac{m_{j}}{\rho_{j}}\;W_{ij}}.$$

The stress tensor is denoted by S in Equation (6) that can be expressed in terms of the pressure, *p*, and the deviatoric stress tensor, $S^{\mathrm{dev}}$,

(10) $$\;S=-pI+S^{\mathrm{dev}},$$

where I is the second order unity tensor. The pressure at particle *i* is calculated by using the following equation of state,

(11) $$\;p_{i}=K\left(\frac{\rho_{i}}{\rho_{i0}}-1\right),$$

where *K* is the bulk modulus and $\rho_{i0}$ is the initial density of the particle *i*. The pressure field may become noisy due to errors introduced in the estimation of density, especially near boundaries and for large deformations. To remedy this issue, we apply a Shepard filter to the density in order to reduce the noise disturbing the solution and, hence, obtain a smooth pressure field. The smoothed density is obtained by

(12) $$\;{\bar{\rho }}_{i}=\frac{\sum_{j=1}^{N}m_{j}W_{ij}}{\sum_{j=1}^{N}\frac{m_{j}}{\rho_{j}}\;W_{ij}}.$$

Colagrossi and Landrini [29] suggested that it is best to perform the density filtering every 20–50 time steps.Therefore, the filtering here is only applied every 40 time steps in order to not adversely alter the density field. The deviatoric stress is determined by using the Jaumann stress rate and assuming that the elastic behavior of the material is linear.

(13) $$\;\frac{dS_{i}^{\mathrm{dev}}}{dt}=2G\left({\dot{E}}_{i}-\frac{1}{3}\mathrm{tr}\left({\dot{E}}_{i}\right)I\right)-S_{i}^{\mathrm{dev}}\;W_{i}+W_{i}\;S_{i}^{\mathrm{dev}},$$

where *G* is the shear modulus. $\dot{E}$ and W are the rate of strain tensor and the spin tensor, respectively, defined by

(14) $$\begin{array}{cc} \;\dot{E} & =\frac{1}{2}\left(\nabla \vec{v}+\nabla \vec{v}{\;}^{T}\right),\\ \;W & =\frac{1}{2}\left(\nabla \vec{v}-\nabla \vec{v}{\;}^{T}\right), \end{array}$$

where $\nabla \vec{v}$ is the velocity gradient tensor,

(15) $$\;\nabla {\vec{v}}_{i}=\sum_{j=1}^{N}\frac{m_{j}}{\rho_{j}}\left({\vec{v}}_{j}-{\vec{v}}_{i}\right)⊗\tilde{\nabla }W_{ij}.$$

In Equation (6), ${\vec{f}}_{ij}{\;}^{\mathrm{HG}}$ is the hourglass force applied in order to prevent the tensile instability and zero-energy modes by penalizing spurious displacements. It is calculated based on the hourglass method proposed by Ganzenmüller et al. [30] for Lagrangian SPH, which was recently applied to Eulerian SPH as well as by Mohseni-Mofidi and Bierwisch [31], which is defined by

(16) $$\;{\vec{f}}_{ij}{\;}^{\mathrm{HG}}=\frac{{\vec{f}}_{ij}{\;}^{\mathrm{HG}{\;}^{\mathrm{stiff}}}+{\vec{f}}_{ij}{\;}^{\mathrm{HG}{\;}^{\mathrm{visc}}}}{2},$$

where ${\vec{f}}_{ij}{\;}^{\mathrm{HG}{\;}^{\mathrm{stiff}}}$ is the stiffness-based hourglass force and ${\vec{f}}_{ij}{\;}^{\mathrm{HG}{\;}^{\mathrm{visc}}}$ is the viscosity-based hourglass force obtained as described in [31]. In Equation (6), ${\vec{f}}_{i}{\;}^{\mathrm{cont}}$ is the contact force acting on particle *i* of a solid body by the nearest particle of another contacting solid body. Section 3 explains how contacts are identified and contact forces are calculated.

Although solid grains are also discretized by SPH particles that carry quantities, such as mass, density and velocity, these constituent particles are moving rigidly together and, therefore, there is no relative movement among them. Consequently, their density does not alter and they do not undergo any deformation. The velocity of the constituent particles of each solid grain is calculated by using a quaternion-based rigid body motion solver proposed by Omelyan [32] and described in the context of SPH by Polfer et al. [33] and Dietemann et al. [34]. The motion of the solid grain *s* is determined according to its initial conditions and the following total force and torque due to solid body contacts,

(17) $$\begin{array}{cc} \;{\vec{F}}_{s} & =\sum_{j=1}^{N^{b}}{\vec{f}}_{j}{\;}^{\mathrm{cont}},\\ \;{\vec{T}}_{s} & =\sum_{j=1}^{N^{b}}{\vec{r}}_{cj}\times {\vec{f}}_{j}{\;}^{\mathrm{cont}}, \end{array}$$

where $N^{b}$ is the number of the boundary particles of the solid grain *s*, ${\vec{r}}_{cj}$ is the distance vector of the boundary particle *j* relative to the center of the grain *s*.

## 3. Solid Bodies Contact

Surface erosion occurs gradually as solid grains hit a surface. Each impact deforms the surface and consequently surface material degradation. After several impacts, the surface material is no longer able to withstand further loading and, therefore, fractures, and is eroded away by a solid grain. Solid grains are considered as rigid bodies that do not experience any deformation or material degradation. A surface is modeled as an elastic–plastic material. The elastic response of the surface is linear. Plastic deformations are calculated by a $J_{2}$ plasticity model in conjunction with a cutting plane return mapping algorithm [35]. The yield stress $\sigma_{y}$ is described by the Johnson–Cook constitutive model [36],

(18) $$\;\sigma_{y}=\left[A+B{\left(\varepsilon_{\mathrm{eff}}^{p}\right)}^{n}\right]{\left[1+\frac{{\dot{\varepsilon }}_{\mathrm{eff}}^{p}}{\dot{\varepsilon_{0}}}\right]}^{C}\left[1-{\left(\frac{T-T_{\mathrm{ref}}}{T_{\mathrm{melt}}-T_{\mathrm{ref}}}\right)}^{m}\right],$$

where $\varepsilon_{\mathrm{eff}}^{p}$ is the equivalent plastic strain, ${\dot{\varepsilon }}_{\mathrm{eff}}^{p}$ is the equivalent plastic strain rate, $\dot{\varepsilon_{0}}$ is a reference plastic strain rate, *T* is current temperature, $T_{\mathrm{ref}}$ is a reference temperature, and $T_{\mathrm{melt}}$ is the melting temperature. Moreover, *A*, *B*, *C*, *n*, and *m* are the model parameters to be determined using experiments.

The damage caused by impacts on to the surface is measured by a scalar damage variable, *D*, which evolves following the Johnson–Cook fracture model [36] as

(19) $$\;\dot{D}=\frac{{\dot{\varepsilon }}_{\mathrm{eff}}^{p}}{\varepsilon_{f}^{p}},$$

where $\varepsilon_{f}^{p}$ is the equivalent plastic strain at fracture determined by

(20) $$\;\varepsilon_{f}^{p}=\left[D_{1}+D_{2}\exp\left(D_{3}\sigma^{★}\right)\right]\left[1+D_{4}\ln\left(\frac{{\dot{\varepsilon }}_{\mathrm{eff}}^{p}}{{\dot{\varepsilon }}_{0}^{f}}\right)\right]\left[1+D_{5}\left(\frac{T-T_{\mathrm{ref}}}{T_{\mathrm{melt}}-T_{\mathrm{ref}}}\right)\right],$$

where $\sigma^{★}=-\frac{p}{\sigma_{\mathrm{eq}}}$ is the stress triaxiality ratio with $\sigma_{\mathrm{eq}}=\sqrt{\frac{3}{2}S^{\mathrm{dev}}:S^{\mathrm{dev}}}$ being von Mises equivalent stress, ${\dot{\varepsilon }}_{0}^{f}$ is the reference fracture strain rate and $D_{1}$ to $D_{5}$ are the material dependent constants. Damage variable *D* is initially zero and increases only with plastic deformation, according to Equation (19). Therefore, *D* for a given particle on the surface grows larger by each solid grain impact until it becomes one indicating fracture of that SPH particle. At this point, the fully damaged particle is removed from the simulation domain.

Solid grains transfer their kinetic energy to the surface upon impact, and as a result deform the surface at the contact interface. Here, contacting surfaces are assumed to be perfectly smooth and ideal. Contact between two solid bodies is determined at the SPH particles level, meaning contact forces are calculated based on particle–particle interactions. In what follows, contact detection and then contact force calculation are explained.

### 3.1. Contact Detection

**Figure 1:**
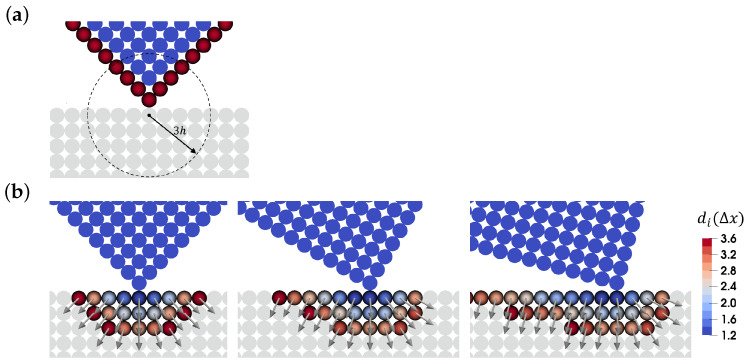
Contact of two solid bodies, where the top one is the primary body and the bottom one is the secondary one. SPH particles are located at the centers of the circles. The circles diameter is the same as Δx that is the particle spacing. (**a**) Boundary particles of the primary surface (red particles) that contribute to the contact calculations of the marked secondary particle with the depicted supporting domain. (**b**) Distance $d_{i}$ and normal vector ${\vec{n}}_{i}$ for the secondary particles.

At any contact, two surfaces are involved, which, here, we distinguish into a primary and a secondary surface. A contact is detected when the distance of a secondary surface particle, hereinafter called secondary particle, from the primary surface, is equal or less than a desired equilibrium distance, which, in this paper, is equal to the initial particle spacing, Δx. The distance of a secondary particle *i* from the primary surface is defined by

(21) $$\;\;d_{i}=\frac{\sum_{j=1}^{N^{\mathrm{pb}}}\left({\vec{n}}_{i}\;\cdot \;{\vec{x}}_{ij}\right)\frac{m_{j}}{\rho_{j}}W_{ij}}{\sum_{j=1}^{N^{\mathrm{pb}}}\frac{m_{j}}{\rho_{j}}\;W_{ij}},$$

where the summation is carried out over the $N^{\mathrm{pb}}$ boundary particles of the primary surface that lies in the supporting domain of the secondary particle *i*, Figure 1a. ${\vec{n}}_{i}$ is the outward normal vector of the primary surface that is realized by the secondary particle *i* and is defined by

(22) $$\;\;{\vec{n}}_{i}=\frac{\sum_{j=1}^{N^{\mathrm{pb}}}\frac{{\vec{x}}_{ij}}{x_{ij}}\frac{m_{j}}{\rho_{j}}W_{ij}}{\sum_{j=1}^{N^{\mathrm{pb}}}\frac{m_{j}}{\rho_{j}}\;W_{ij}}.$$

Using Equation (22), surface discontinuities, such as sharp corners, are smoothed out, resulting in a smooth transition from one side of a discontinuity to the other side. As described in Figure 1, the normal vector of the sharp corner of the primary surface that is realized by the secondary particles changes smoothly as the primary solid body rotates. Although the primary surface normal vector changes locally, the normal vector realized by the closest secondary particle to the corner stays almost unchanged, which results in a correct estimation of distance $d_{i}$. Additionally, it can be observed that due to the smoothing nature of Equations (21) and (22), the distance between two touching particles is estimated a bit larger than a given particle spacing. The smoothing effect can be adjusted by using different kernels for the contact model or altering the size of the support domain of secondary particles. However, throughout this paper we use the same kernel with the fixed smoothing length, which leads to accurate modeling of contacts between solid bodies.

Having obtained the normal distance of all secondary particles from the primary surface, contacts are detected by determining whether a secondary particle has penetrated the primary surface or not. It is done by calculating the penetration for secondary particles *i* as

(23) $$\;\;P_{i}=\Delta x-d_{i}.$$

A contact is then identified for a secondary particle *i* only if $P_{i}$ is positive.

### 3.2. Contact Force Calculation

To prevent contacting surfaces from penetration, as soon as a contact is detected at a secondary particle *i*, a contact repulsion force parallel to the surface normal vector, ${\vec{n}}_{i}$, is calculated by using a constant linear spring repulsion model as

(24) $$\;\;{\vec{f}}_{i}{\;}^{r}=k_{r}P_{i}{\vec{n}}_{i},$$

where $k_{r}$ is the repulsion spring stiffness. The secondary particle *i* is in contact with the primary surface, as long as $P_{i}$ is positive.

A contact friction force is determined by using the method proposed by Cundall and Strack [37] originally for DEM. In this method, the friction force is integrated during the time of contact. Vyas et al. [38] recently showed that introducing such a method to SPH calculations of contact friction forces is essential to correctly predict a contact’s behavior. Following this method, the secondary particle *i* is connected to the nearest primary particle *j* with a fictitious friction spring that is initially unloaded with zero displacement, $|\Delta {\vec{l}}_{i}|=0$. The spring is loaded with the relative movement of particles *i* and *j* in the contact plane for which ${\vec{n}}_{i}$ is the normal vector. The contact friction force is then calculated proportional to the spring displacement, which is integrated over the contact time as

(25) f→ifn+1=−kfΔl→i(n+1=−kfΔl→i(n+v→ij(n+1Δt·t→i(n+1t→i(n+1,

where Δt is the simulation time step, ${\vec{v}}_{ij}={\vec{v}}_{i}-{\vec{v}}_{j}$ is the relative velocity of the secondary particle *i* and its nearest primary particle *j*. The superscripts n+1 and *n* indicate the value of a quantity for the current time step and for the previous one, respectively. In addition, ${\vec{t}}_{i}$ is the tangential vector of the contact plane at the secondary particle *i* that is defined by

(26) $$\;\;{\vec{t}}_{i}=\frac{{\vec{x}}_{ij}-{\vec{n}}_{i}\;\left({\vec{x}}_{ij}\;\cdot \;{\vec{n}}_{i}\right)}{\left|{\vec{x}}_{ij}-{\vec{n}}_{i}\;\left({\vec{x}}_{ij}\;\cdot \;{\vec{n}}_{i}\right)\right|}.$$

Moreover, in order to impose the sliding friction condition, the contact friction force is limited by the Coulomb friction force, resulting in the following final expression for the contact friction force.

(27) $$\;\;{\vec{f}}_{i}{\;}^{f}=\min\left(\mu \;|{\vec{f}}_{i}{\;}^{r}\left|,\;\right|{\vec{f}}_{i}{\;}^{f}|\right)\frac{{\vec{f}}_{i}{\;}^{f}}{|{\vec{f}}_{i}{\;}^{f}|},$$

where μ is the coefficient of friction. Finally, the total contact force acting on the secondary particle *i* is obtained as

(28) $$\;\;{\vec{f}}_{i}{\;}^{\mathrm{cont}}={\vec{f}}_{i}{\;}^{r}+\;{\vec{f}}_{i}{\;}^{f}.$$

Additionally, a contact force of the same magnitude, but in the opposite direction, is also applied to the primary particle *j*,

(29) $$\;\;{\vec{f}}_{j}{\;}^{\mathrm{cont}}=-\;{\vec{f}}_{i}{\;}^{\mathrm{cont}}.$$

### 3.3. Results and Verification

Three test cases, namely free sliding on a slope, controlled sliding on a flat surface, and, Hertzian contact of a half cylinder and a flat rigid surface, are considered here to examine the accuracy of the method employed in this paper to handle solid bodies contact.

#### 3.3.1. Free Sliding on a Slope

**Figure 2:**
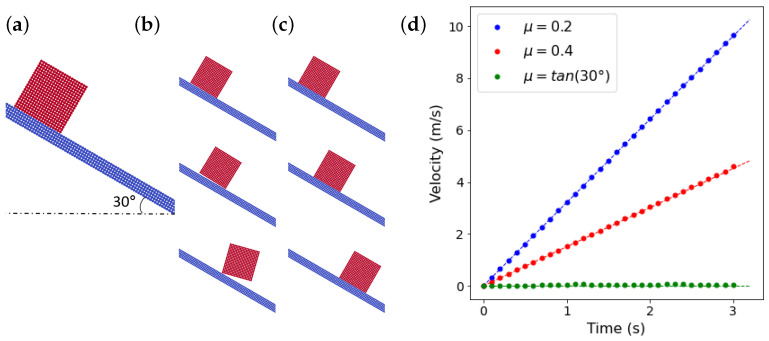
(**a**) The initial two-dimensional model where the slider (in red) is the primary solid body and (**b**) snapshots of a simulation where the contact model is only based on particle-particle penetration which leads to wrong solutions. (**c**) Snapshots of a simulation where the new contact model is employed. Both sets of snapshots are taken at 0.5 s, 1.0 s and 1.5 s, from top to bottom. (**d**) The velocity of the slider parallel to the slope, dots represent the simulations results and dash-lines represent the analytical solutions of this problem.

A rigid square slider, considered here the primary body, is placed on a rigid slope with 30° inclination, Figure 2a. The slider should ideally slide under gravity smoothly down the slope. In most particle-based methods, contact forces are calculated based on particle-particle penetrations, without using any geometrical information of the contacting surfaces. Therefore, flat smooth surfaces are modeled with an artificial roughness in the order of the particle spacing, which results in a wrong movement for the slider as demonstrated in Figure 2b. However, as shown in Figure 2c, by using the current contact model, the slider moves correctly down the slope as expected. Figure 2d shows the comparison between the following analytical solutions and the simulation results for the velocity of the slider parallel to the slope, $v{\;}^{\mathrm{slider}}$, with different coefficients of friction,

(30) $$\;\;v{\;}^{\mathrm{slider}}=\left(\sin\theta -\mu \cos\theta \right)gt,$$

where θ is the inclination angle of the slope, *t* is time and *g* is the acceleration due to gravity. The results are in very good agreement, proving that the present contact algorithm not only correctly models a smooth surface, but also models friction accurately.

#### 3.3.2. Controlled Sliding on a Flat Surface

**Figure 3:**
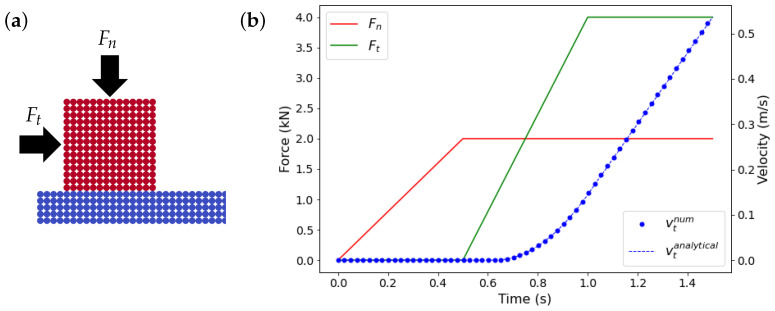
The initial two dimensional model where the slider, in red, is the primary solid body (**a**). The velocity of the slider parallel to the slope (**b**), dots represent the simulations results, and dashed-lines represent the analytical solutions of this problem.

A rigid slider slides on a rigid flat surface, where its movement is controlled by a normal force $F_{n}$ and a tangential force $F_{t}$ applied externally, Figure 3a,b shows the time evolution of the external forces. The coefficient of friction is equal to 0.5 and, therefore, according to the Coulomb friction model the slider must stay stationary for $F_{t}$ less than $0.5\;F_{n}$. At time 0.625 s when $F_{t}$ is equal to $0.5\;F_{n}$, the slider must start sliding with an increasing acceleration until 1 s and, after that, keep moving with a constant acceleration. The calculated tangential velocity of the slider is compared with the analytical solution in Figure 3, where a very good agreement between the results is observed.

#### 3.3.3. Hertzian Contact of a Half Cylinder and a Flat Rigid Surface

**Figure 4:**
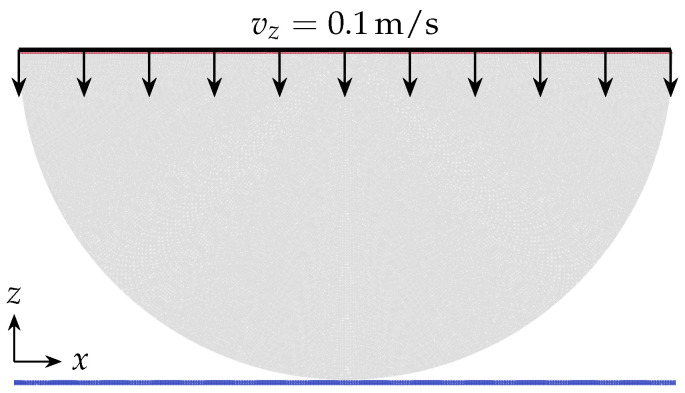
Initial geometry of the Hertzian contact test case where the upper boundary of the cylinder is moving downward with a constant velocity.

Contact of an elastic cylinder with a flat rigid surface is investigated. The cylinder is made of aluminum with elastic modulus of 70 GPa and Poisson’s ratio of 0.3. Half of the cylinder is modeled in 2D, Figure 4, assuming that the stress state is plane strain. The radius of the cylinder is 25 mm. The analytical solution of this problem can be found by considering the Hertz contact theory, provided that the rigid flat surface is a cylinder with an infinite elastic modulus and radius. The half-width of the contact area *a* and the maximum contact pressure $p_{0}$ are defined by

(31) $$\begin{array}{cc} \;a & =2{\left(\frac{QR}{\pi E}\right)}^{\frac{1}{2}},\\ \;p_{0} & ={\left(\frac{QE}{\pi R}\right)}^{\frac{1}{2}}, \end{array}$$

where *Q* is the applied load per unit length and, *E* and *R* are the elastic modulus and radius of the cylinder, respectively. Additionally, the pressure distribution along the contact-line in *x* direction reads

(32) $$\;p\left(x\right)=p_{0}\sqrt{1-{\left(\frac{x}{a}\right)}^{2}}.$$

**Figure 5:**
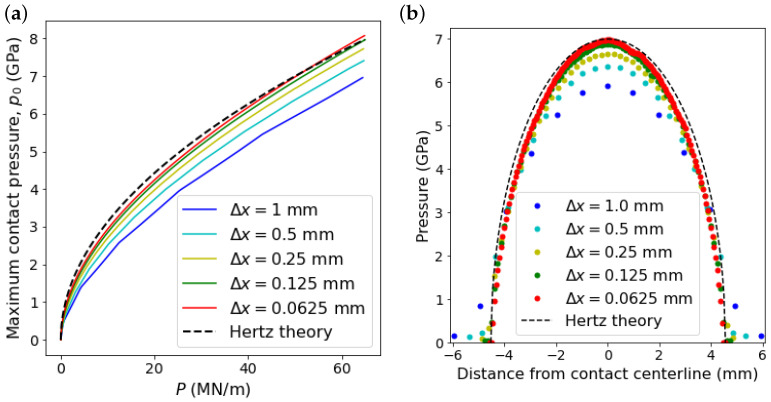
Comparison between the calculated contact pressure for different particle spacings and the Hertz theory; (**a**) maximum pressure at contact in terms of the applied load and (**b**) contact pressure distribution along the contact line for the applied load of 50 MN/m.

**Figure 6:**
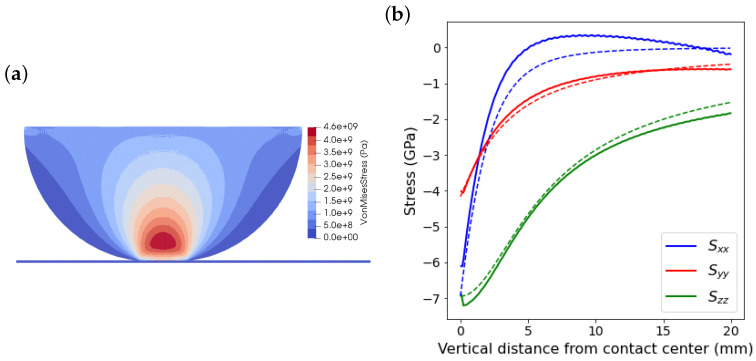
Numerical results for (**a**) von Mises stress field and (**b**) comparison between the calculated stress fields along the axis of symmetry and analytical solutions for the applied load of 50 MN/m with particle spacing of 0.0625 mm, where solid lines are the numerical results and dashed lines are the analytical solutions.

Here, the upper boundary of the half-cylinder moves with a constant velocity of 0.1 m/s, Figure 4. The applied normal load corresponding to the movement of the upper boundary is defined during the simulation by calculating the reaction force of the rigid surface, which increases with the boundary’s movement. Figure 5a shows the maximum contact pressure in terms of the applied load obtained from simulations for different particle spacings and from Equation (31). The results show the convergence of the numerical estimations to the Hertz theory as the resolution of the particle distribution increases. In Figure 5b, the estimated pressure distribution along the contact line, in *x* direction, for the applied load of 50 MN/m is presented and compared with the analytical solution. It can be observed that the pressure calculated at boundary particles of the cylinder approaches the analytical solution, Equation (32), by decreasing the particle spacing. In addition, the obtained stress fields are examined. Figure 6a shows the von Mises stress obtained for the applied load of 50 MN/m. Moreover, the calculated stress fields are evaluated on the axis of symmetry of the cylinder and compared with the analytical solutions reported by Tripp [39], which are expressed as

(33) $$\begin{array}{cc} \;S_{xx} & =-\frac{p_{0}}{{\left(1+\zeta^{2}\right)}^{1/2}}\left[2\zeta^{2}+1-2\zeta {\left(1+\zeta^{2}\right)}^{1/2}\right]\\ \;S_{zz} & =-\frac{p_{0}}{{\left(1+\zeta^{2}\right)}^{1/2}}\\ \;S_{yy} & =\nu \left(S_{xx}+S_{zz}\right) \end{array}$$

where ν is Poisson’s ratio and $\zeta =d_{z}/a$ is a non-dimensional parameter with $d_{z}$ being the vertical distance of a material point on the axis of symmetry from the contact center. The stress fields are presented in Figure 6b, which show a very good agreement with the analytical solutions.

## 4. Surface Erosion Modeling

**Figure 7:**
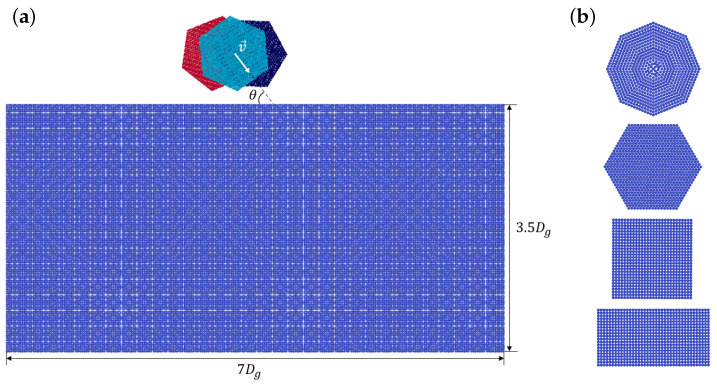
(**a**) Initial geometry employed to study the surface erosion due to solid grains impacts and (**b**) grain shapes, namely octagon, hexagon, square, and rectangle (**top** to **bottom**).

Surface erosion is defined as the total mass loss of a surface per total mass of the impacting solid grains. It is a common practice to define a surface erosion, in terms of impact angle for a given type of grains, and a given impact velocity. Therefore, to study the erosion behavior of a surface, blasting tests are usually employed where a large number of grains impact a single surface with a given nominal impact velocity and impact angle. Modeling such a process in full scale is not computationally possible, because the domain and number of impacts are too large. Therefore, alternatively, surface erosion is modeled by impacts of a reasonable number of single grains hitting the surface successively. Li et al. [23] studied the effect of overlapping impacts on the surface erosion. They showed that the surface damage accumulation may considerably vary depending on the distance between two consecutive impacts. In the present study, therefore, in order to take the effect of overlapping impacts into account, the solid particles are positioned randomly within a given range while their distance from the surface is fixed. In addition, in order to consider all possible impact conditions, the grains are randomly oriented. Figure 7a shows the 2D model used in this paper to numerically study the erosion behavior of stainless steel AISI 304 due to impacts of angular solid grains. As already mentioned, the steel surface is initially modeled perfectly smooth without any roughness. The same assumption is applied to the surface of the grains and, therefore, the flat sides of the grains are ideally smooth. The surface dimensions are chosen, such that, according to Leguizamón et al. [26], the impact results are independent of the size of the numerical model. The particle spacing, Δx, is set to 8 μm for all impact simulations, which results in about 32 SPH particles per grain diameter. That guarantees accurate calculations based on the resolution study carried out by Leguizamón et al. [26]. The mechanical and physical parameters of the surface are reported in Table 1. In this study, we use angular grains that have the material properties of glass (ρ=2500 kg/$m^{3}$), but are unbreakable and non-erodible. The grains have the diameter, $D_{g}$, of 250 μm and hit the surface with the impact velocity of 100 m/s. The grains are modeled as rigid clusters of SPH particles in four different shapes shown in Figure 7b. All grains have almost the same area that is equal to the area of a circle with the diameter of 250 μm.

**Figure 8:**
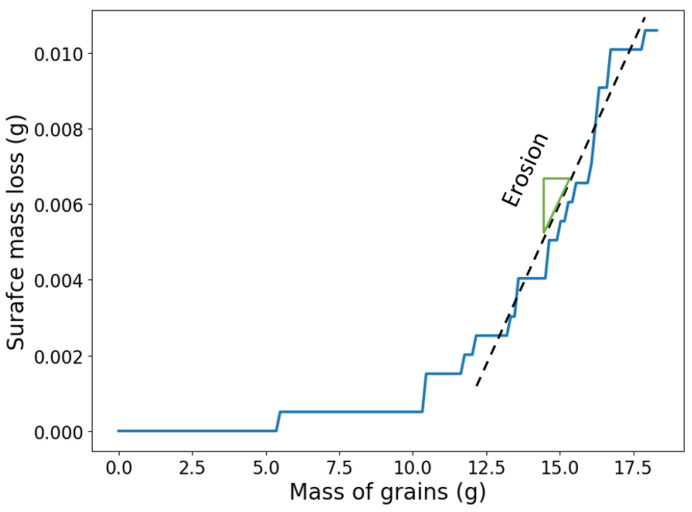
Surface mass loss growth in terms of the mass of the grains for impact angle of 15° and one surface sample. Erosion is calculated as the slope of the dashed line fitted to the steady part of the mass loss growth.

Each impact is modeled by a single simulation and, then the next impact is carried out by removing the old grain, adding a new one that travels toward the surface in the subsequent simulation. We assume that the change of surface temperature between subsequent impacts is negligible and, hence, the temperature is reinitialized to the room temperature for each new simulation. After each impact, the mass loss is determined by identifying the number of failed particles that have been removed from the simulation. As a result, we are able to define the mass loss evolution in terms of the mass of the solid grains. Figure 8 shows the results obtained after 140 impacts of hexagonal grains at impact angle of 15°. During the incubation period, the impacts cause no surface mass loss and only strain the surface and increase the damage. After the incubation period, surface mass loss grows as the number of impacts increase. After a certain amount of grain mass, the growth rate of the mass loss, in terms of the grain mass, becomes almost constant and, hence, is considered as the surface erosion for an impact angle of 15°.

**Figure 9:**
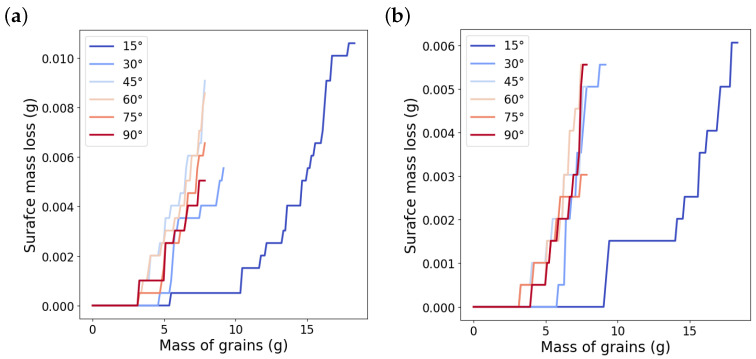
Mass loss development of two surface samples (**a**,**b**) in terms of the mass of the grains for different impact angles.

**Figure 10:**
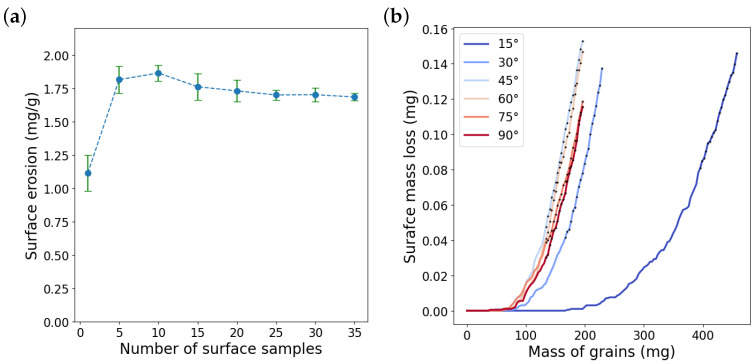
(**a**) Convergence of surface erosion in terms of the number of surface samples for impact angle of 45° and (**b**) mass loss obtained by averaging the results for 25 surface samples. These results are obtained for hexagonal grains with the distribution range of $1.0D_{g}$. The black marks represent the data points used to estimate erosion.

It is well known that surface erosion varies with impact angle. Therefore, in order to predict the erosion behavior of the surface, erosion is calculated by impact simulations with different impact angles from 15° to 90° with 15° intervals. The number of impacts must be large enough so that we obtain an approximately linear rate of the surface mass loss. Since the mass loss increases with a different rate for different impact angles, the required number of impacts may vary with the impact angle. Here, for the hexagonal grains, we found 140 impacts for 15°, 70 impacts for 30°, and 60 impacts for the other angles suffice. For each impact angle, an intact surface is hit by the number of grains mentioned above. Each surface may be regarded as a small surface sample of a specimen used in a blasting test that is eroded by a set of random impacts. Figure 9a shows the surface mass loss in terms of the mass of the grains calculated for a surface sample for all impact angles. As the specimen in a blasting test is hit by different sets of random impacts at locally different spots across the surface, we redo the calculations for new surface samples with new sets of random positions and orientations. The results for a second sample are reported in Figure 9b. It was observed that, although impact simulations on one surface sample results in certain mass loss rates at different angles, they do not represent the whole statistics of all possible impacts. Therefore, impact simulations are carried out for several surface samples in order to attain sufficient ensemble averaging. Figure 10a shows the surface erosion calculated for the impact angle of 45° by using different numbers of surface samples. To determine the surface erosion, only the last 20 data points of the mass loss evolution are considered to calculate the average erosion and standard deviation. For surface sample numbers larger than 25, average erosion does not vary considerably. Moreover, in general, the standard deviation decreases by increasing the number of surface samples and is marginal for sample numbers greater than 25. Hence, hereinafter, we use 25 surface samples to obtain surface erosion. Figure 10b demonstrates the surface mass loss evolution obtained by averaging the results for 25 surface samples. For all impact angles, we observe an incubation period followed by a nonlinear mass loss growth until it becomes rather steady and linear as a function of the mass of impacting grains.

**Figure 11:**
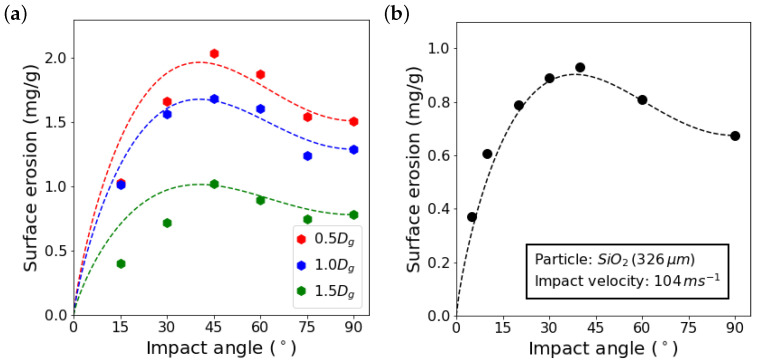
(**a**) Surface erosion as a function of the impact angle for three different ranges of grain center distributions; dots are the calculated erosions and dashed lines are the predictions obtained by Oka’s erosion model. (**b**) Surface erosion of stainless steel AISI 304 obtained by blasting tests reported by Oka et al. [13].

As already mentioned, overlap of impacts plays an important role in the numerical predictions of surface erosion behavior. By randomly positioning the grains, we try to statistically approximate all possible overlapping events. The grains are randomly positioned within a given range. By altering this range, we obtain different sets of impacts in which the number of overlapping impacts are considerably different, as well as the overlapping distances. Here, we employ three different ranges of $0.5D_{g}$, $1.0D_{g}$, and $1.5D_{g}$. For example, by using the range of $1.0D_{g}$, the center of the grains for impact angle of 90° is randomly defined between $-0.5D_{g}$ and $0.5D_{g}$, relative to the symmetry axis of the surface. For any other impact angle, grains are shifted accordingly so that they hit the surface around the midpoint of the surface. Figure 11a shows erosion calculated for each distribution range by averaging the simulation results of impacts of 25 surface samples with hexagonal grains. The calculated values are compared with predictions obtained by using Oka’s erosion model,

(34) $$\;E\left(\alpha \right)=g\left(\alpha \right)E_{90},$$

where α is the impact angle, $E_{90}$ is the erosion at impact angle 90° and g(α) is an angle dependent function that defines the erosion behavior of the surface and expressed by

(35) $$\;g\left(\alpha \right)={\left(\sin\alpha \right)}^{n_{1}}{\left(1+\mathrm{Hv}\left(1-\sin\alpha \right)\right)}^{n_{2}},$$

where Hv is the hardness number of the surface, which is the Vickers hardness normalized by 1 GPa, and $n_{1}$ and $n_{2}$ are model parameters that are determined by the surface hardness and grain properties. Here, first, we define g(α) according to the parameters reported by Oka et al. [13] for stainless steel AISI 304 and, then, multiply it by the calculated $E_{90}$ for each distribution range to obtain Oka’s predictions, reported in Figure 11a by dashed lines. According the measurements for surface erosion of AISI 304 by Oka et al. [13], presented in Figure 11b, erosion increases with impact angle up to 40° and then gradually decreases as the impact angle becomes larger. The calculated erosion shows almost the same behavior for all distribution ranges. However, the obtained results for the distribution range of $1.0D_{g}$ are in the best agreement with the Oka’s prediction over the whole range of impact angles. On the one hand, decreasing the range from $1.0D_{g}$ to $0.5D_{g}$ results in a higher number of overlapping impacts with smaller overlapping distance. This intensifies the effect of overlapping impacts and hence increases the erosion. However, surface erosion at impact angles 15° and 30° does not increase considerably, which results in a rather large deviation of the results from the Oka’s prediction at low impact angles. On the other hand, increasing the distribution range from $1.0D_{g}$ to $1.5D_{g}$ reduces the surface erosion considerably, as the number of overlapping impacts decrease drastically and the overlapping distance increases as well. Although the results are in agreement with Oka’s prediction at large impact angles, there is a large discrepancy at low impact angles. Therefore, the results show that, by using the distribution range of $1.0D_{g}$, we can realize a correct prediction of the surface erosion behavior over the whole range of impact angles, which gives us confidence in the reliability of the developed methodology.

**Figure 12:**
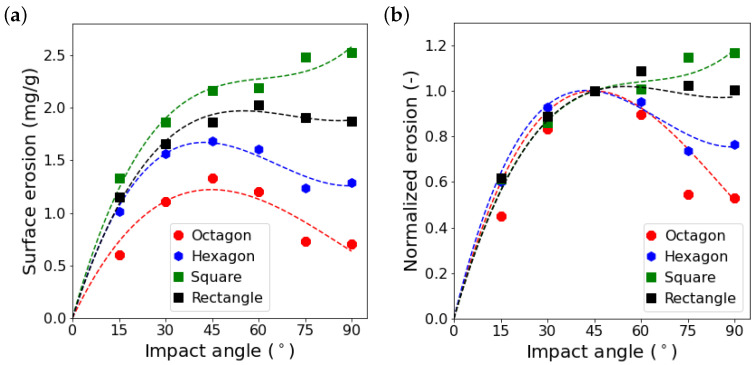
Surface erosion (**a**) and normalized erosion (**b**) for different grain shapes. Surface erosion is divided by the erosion at an impact angle of 45° in order to calculate the normalized erosion. The dashed lines are third order polynomials fitted to the simulated values represented by the symbols.

**Figure 13:**
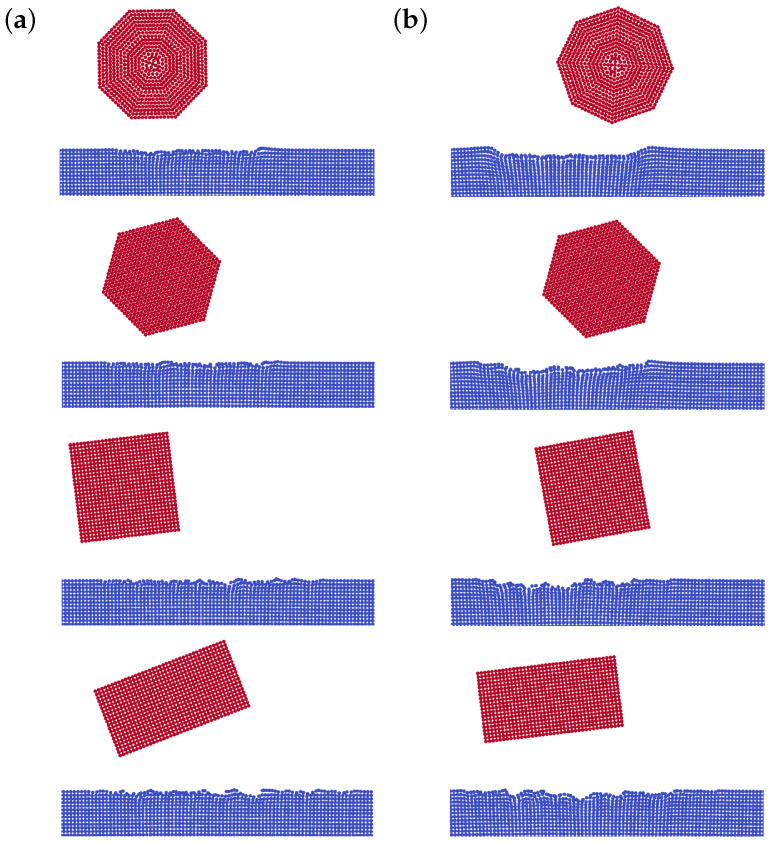
Eroded surface after 50 impacts for impact angle of (**a**) 30° and (**b**) 90°.

It is worth mentioning that Oka et al. [13] used irregularly shaped grains in their experiments, but here we could reproduce the erosion behavior by simply using regular hexagonal grains. By using the presented numerical methodology, we are now able to study the effect of different parameters, such as grain size, grain shape, and impact velocity. Here, we do a simple parameter study in order to investigate the influence of the grain shape on the surface erosion behavior. Among all important factors, we picked grain shape because it is the hardest one to study by experiments. In addition to hexagonal grains, we analyze the surface erosion for octagonal, square, and rectangular grains, as reported in Figure 12a. The grains are characterized by two shape factors, angularity $\left(A_{n}\right)$ and aspect ratio $\left(e\right)$. Here, we adopt the definition proposed by Palasamudram and Bahadur [43] for angularity that is calculated by

(36) $$\;A_{n}=\sqrt{2\pi }\sum_{i=1}^{n}a_{i}p_{i},$$

where *n* is the number of corners, $a_{i}=\sqrt{2\pi }/\beta_{i}$ is the angularity of corner *i* with $\beta_{i}$ as the corner angle in radians and $p_{i}=\left(\pi -\beta_{i}\right)/2\pi$ is the probability of corner *i* to hit the surface. In addition, aspect ratio is defined as the ratio of the maximum Feret diameter of a grain to its minimum Feret diameter. Angularity and aspect ratio of the studied grains are reported in Table 2. Both angularity and aspect ratio increase as the number of edges decreases, except that the aspect ratio for a rectangle is higher than for a square, although they both have the same number of edges and angularity. In general, the more angular the grains are, the more erosion is caused by them. However, the functional dependency on the impact angle changes if the grain shape is modified. The erosion behavior for all grain shapes is almost the same up to impact angles of 45°, as shown in Figure 12b. Up to an impact angle of 45°, the dominant erosion mechanism is “cutting”, as shown in Figure 13a, where it can be observed that the surface deformation is not considerable. It can also be seen that the surface is still rather smooth and erodes uniformly. However, for impact angles larger than 45°, as demonstrated in Figure 13b, the surface roughens considerably. The normal impact force is large enough to deform the surface and grains with sharp corners can penetrate the surface and, consequently, alter its morphology. The impact conditions, specifically the actual impact angle, vary as the surface roughness changes, which results in additional erosion due to cutting, in addition to surface deformation and fatigue. Therefore, the effect of the angularity is the strongest for square and rectangular grains at an impact angle of 90°, for which the surface after 50 impacts is much rougher than the surface for octagonal and hexagonal grains (cf. Figure 13b). This manifests itself, as demonstrated by Figure 12b, in the strong dependence of the surface erosion behavior on the grain shape for large impact angles. Similar observations were made by Liu et al. [24] who studied the effect of grain shape by using 3D finite element simulations. Nonetheless, comparing the results for square and rectangular grains, which have the same angularity, we observe that the rectangular grains are less erosive than the square ones. This can be explained by the fact that grains with a larger aspect ratio are more likely to rotate after hitting a surface. This behavior causes less surface damage as some part of the translational kinetic energy of the impact is converted to rotational kinetic energy [20]. It can be seen in Figure 13 that the higher the aspect ratio of the grains, the wider the region that the grains attack due to second impacts because of rotation.

**Figure 14:**
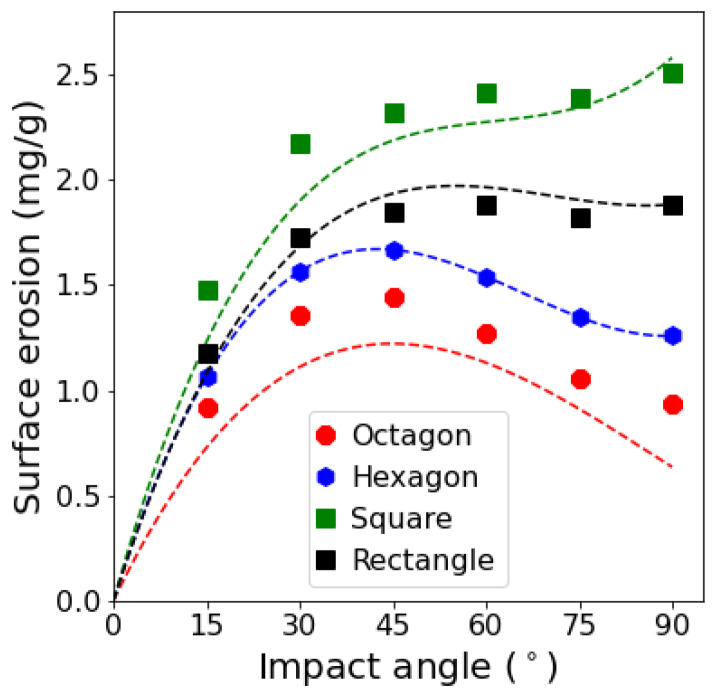
Comparison between the simulation results and the erosion estimations of the modified Oka’s model, Equation (37). The dashed lines represent the third order polynomials fitted to the simulation results shown in Figure 12 and dots are the erosion estimations of the modified Oka model.

Based on the results mentioned above, we modify the Oka’s erosion model, Equation (34), in order to incorporate the effect of grain shape in terms of angularity and aspect ratio. We take the hexagonal grain shape as the reference grain shape since it leads to the closest numerical prediction of the erosion behavior of stainless steel AISI 304 in comparison to Oka’s findings. We introduce a modification function $h(e,A_{n},\alpha )$ to Oka’s model as

(37) $$\;E\left(\alpha \right)=g\left(\alpha \right)h\left(e,A_{n},\alpha \right)E_{90},$$

where g(α) and $E_{90}$ are defined by fitting Oka’s original model to the simulation results for the hexagonal grains. In order to define the modification function, we assume that the function *h* is dependent on impact angle α only at large impact angles greater than 45°, and that erosion increases with angularity and decreases with the aspect ratio. Finally, the proposed modification function is defined by

(38) $$\;\;h\left(e,A_{n},\alpha \right)={\left(\frac{e}{e^{\mathrm{ref}}}\right)}^{n_{3}}{\left(\frac{A_{n}}{A_{n}^{\mathrm{ref}}}\right)}^{n_{4}}\left\{\begin{array}{cc} 1, & \alpha \leq 45^{\circ },\\ {\left(\frac{e}{e^{\mathrm{ref}}}\right)}^{n_{5}\left(\alpha -45^{\circ }\right)}{\left(\frac{A_{n}}{A_{n}^{\mathrm{ref}}}\right)}^{n_{6}\left(\alpha -45^{\circ }\right)}, & \alpha >45^{\circ }, \end{array}\right.$$

where $e^{\mathrm{ref}}$ and $A_{n}^{\mathrm{ref}}$ are the reference aspect ratio and angularity, respectively, which, here, are set to the aspect ratio and angularity of the hexagonal grains and, $n_{3}$, $n_{4}$, $n_{5}$ and $n_{6}$ are the new additional model parameters. Figure 14 shows the comparison between the simulation results, presented in Figure 12, and the estimations of the newly proposed model, Equation (37), where $n_{3}$ to $n_{6}$ are set to −0.5, 1.5, −0.003 and 0.03, respectively. The estimations show a good agreement with the numerical predictions. The modification function is able to correctly produce the erosion behavior for grains of different shapes proving the validity of the assumptions made. Additionally, by considering the values determined for the model parameters $n_{3}$ to $n_{6}$, we can conclude that the effect of angularity on erosion behavior is bigger than the effect of the aspect ratio. However, the influence of the aspect ratio must not be ignored because otherwise the modification function would result in the same erosion estimations for square and rectangular grains.

## 5. Conclusions

In this study, we aimed to numerically study SPE of ductile materials commonly ascribed to the cutting mechanism at shallow impact angles and the deformation mechanism at large angles. In order to do that, we employed SPH and developed a new contact model that can robustly handle contacts around sharp corners by extrapolating grain surface normals onto the material surface to be eroded. In addition, we improved the modeling methodology used by Leguizamón et al. by making sure that sufficient statistically relevant impacts were realized in our simulations, in order to achieve reliable erosion predictions. Although we used 2D models, the methodology can be easily extended to 3D modeling as well.

First, the simulation results show that the improved methodology is able to correctly model the erosion behavior of stainless steel AISI 304 in comparison with the experimental results reported on in the literature. Second, we studied the effect of grain shape on the erosion behavior by using four different uniform polygonal grain shapes. The grains were characterized by two shape factors, namely angularity and aspect ratio. The results show that the higher the angularity, the larger the erosion. Moreover, we observed that the aspect ratio affects the erosion, since grains with a larger aspect ratio are more likely to rotate after impact, which results in less energy absorption by the surface and, hence, less erosion. Finally, based on the numerical findings, we introduced a modification to Oka’s erosion model in order to incorporate the effect of grain shape. The estimations of the modified erosion model are in good agreement with the numerical predictions, showing the predictive quality of the model.

## Figures and Tables

**Table 1:** Material properties for stainless steel AISI 304 [40,41,42].

Material Properties	Symbol	
Density	ρ $\left(\mathrm{kg}/m^{3}\right)$	7890
Elastic modulus	*E* (GPa)	200
Possion’s ratio	ν (–)	0.3
Heat capacity	$C_{P}$ (J/kg-K)	510
*Plasticity model:*		
Yield Stress	*A* (MPa)	555.5
Hardening coefficient	*B* (MPa)	1379
Strain hardening exponent	*n* (–)	0.9497
Strain rate hardening constant	*C* (–)	0.01503
Reference strain rate	$\dot{\varepsilon_{0}}$ $\left(s^{-1}\right)$	0.0001
Temperature softening exponent	*m* (–)	1.3263
Reference temperature	$T_{\mathrm{ref}}$ (K)	293
Melting temperature	$T_{\mathrm{melt}}$ (K)	1673
*Failure criterion:*		
Damage constant	$D_{1}$ (–)	−0.7786
Damage constant	$D_{2}$ (–)	2.5588
Damage constant	$D_{3}$ (–)	−0.3607
Damage constant	$D_{4}$ (–)	−0.0191
Damage constant	$D_{5}$ (–)	0.0
Reference fracture strain rate	${\dot{\varepsilon_{0}}}^{f}$ $\left(s^{-1}\right)$	0.001

**Table 2:** Angularity and aspect ratio of the grains.

Grain Shape	*e*	$A_{n}$
Octagon	1.082	2.67
Hexagon	1.155	3.0
Square	1.414	4.0
Rectangle	2.236	4.0

## Data Availability

The data presented in this study are available upon request from the corresponding author.
